# Survival of *Burkholderia pseudomallei* and Pathogenic *Leptospira* in Cola, Beer, Energy Drinks, and Sports Drinks

**DOI:** 10.4269/ajtmh.19-0948

**Published:** 2020-04-06

**Authors:** Vanaporn Wuthiekanun, Premjit Amornchai, Sayan Langla, Nicholas J. White, Nicholas P. J. Day, Direk Limmathurotsakul

**Affiliations:** 1 Mahidol Oxford Tropical Medicine Research Unit, Faculty of Tropical Medicine, Mahidol University, Bangkok, Thailand;; 2 Centre for Tropical Medicine and Global Health, University of Oxford, Oxford, United Kingdom;; 3 Department of Tropical Hygiene, Faculty of Tropical Medicine, Mahidol University, Bangkok, Thailand

## Abstract

*Burkholderia pseudomallei* and pathogenic *Leptospira* in contaminated drinking water can cause melioidosis and leptospirosis, respectively. Here, we evaluated their survival in beverages. We mixed six isolates (three isolates per organism) in four beverages (Coca-Cola^®^, Red Bull^®^, Singha^®^ beer, and Gatorade^®^) and distilled water as the control at two final concentrations (1 × 10^7^ colony-forming units [CFU]/mL and 1 × 10^3^ CFU/mL). The solution was kept at two temperatures (37°C and 4°C). At 4°C and at the high concentration, pathogenic *Leptospira* survived in Coca-Cola^®^ up to 3 minutes and in Singha, Red Bull^®^, and Gatorade up to 15 minutes, whereas *B. pseudomallei* survived in these beverages up to 8 hours, and 14, 14, and 28 days, respectively. The survival time of both organisms was shorter at 37°C (*P* = 0.01) and at the lower concentration (*P* = 0.001). In conclusion, *Leptospira* can survive in some beverages for up to 15 minutes, whereas *B. pseudomallei* can survive in some beverages for up to 4 weeks.

*Burkholderia pseudomallei* is a Gram-negative environmental bacterium and the causative organism of melioidosis, which kills an estimated 89,000 people per year worldwide.^1^ Humans acquire melioidosis by ingestion, inhalation, or skin inoculation.^2,3^ *B. pseudomallei* is capable of surviving hard environmental conditions, including prolonged nutrient deficiency in distilled water for more than 16 years.^4^ Two outbreaks of melioidosis due to contaminated water supplies have been reported in Australia,^2^ and melioidosis caused by *B. pseudomallei* in drinking water have also been reported in Thailand.^3^

Pathogenic *Leptospira* spp. is a spirochete bacterium and the causative organism of leptospirosis, which kills an estimated 58,900 people per year worldwide.^5^ Humans acquire leptospirosis by drinking or contacting with water, soil, or food contaminated with the urine of infected animals. Pathogenic *Leptospira* remain viable in fresh water for up to 20 months.^6^ A number of outbreaks of leptospirosis have been linked to contaminated drinking water supply in multiple countries, including Chile, India, and Italy.^7–9^ Although *B. pseudomallei* and *Leptospira* spp. could survive in water for a long period of time, it is unclear how long both organisms can survive in common commercial drinks. If containers of drinks have holes or tears, drinks are contaminated with soil (and *B. pseudomallei*) or rat urine (and *Leptospira* spp.), and the organisms can survive for a long period of time, consuming contaminated drinks could lead to infection. In this study, we evaluated survival of *B. pseudomallei* and *Leptospira* spp. in four beverages at 4°C and 37°C.

Isolates used in our study included one environmental *B. pseudomallei* isolate (E8),^10^ two clinical *B. pseudomallei* isolates (NR-9910 and NR-8071), and three pathogenic *Leptospira* isolates (*L. interrogans* serovar Autumnalis [NR-20161], *L. kirschnerii* serovar Grippotyphosa [NR-20327], and *L. interrogans* serovar Pyrogenes [NR-20157]).^11,12^ Survival of these isolates were evaluated in a cola drink (Coca-Cola^®^ original), beer (Singha^®^ original), an energy drink (Red Bull^®^ Extra), and a sports drink (Gatorade^®^ lemon lime) with distilled water used as the control.

*B. pseudomallei* isolates were recovered from frozen vials stored at −80°C, transferred to Columbia agar, and incubated at 37°C for 24 hours. *B. pseudomallei* colonies were harvested, suspended into sterile water, and adjusted to obtain a concentration of about 1 × 10^8^ colony-forming units (CFU)/mL. *Leptospira* isolates were recovered from *Leptospira* Vanaporn Wuthiekanun (LVW) agar stored at room temperature (25–30°C).^13^ Each *Leptospira* strain was subcultured to Ellinghausen and McCullough modified Johnson and Harris (EMJH) broth and adjusted to an optical density of 0.32 at 420 nm to obtain a concentration of about 1 × 10^8^ CFU/mL.

We evaluated the survival of the two organisms in four beverages with two different final concentrations and at two temperatures. Each isolate, at concentrations of about 1 × 10^8^ and 1 × 10^4^ CFU/mL in 0.3 mL of sterile water for *B. pseudomallei* and EMJH broth for *Leptospira*, was inoculated into 2.7 mL (total 3 mL) of each beverage to obtain final concentrations of about 1 × 10^7^ and 1 × 10^3^ CFU/mL. The solutions were mixed and kept in sterile 5-mL tubes. One set of the high concentration (1 × 10^7^ CFU/mL) and one set of the low concentration (1 × 10^3^ CFU/mL) were kept at 4°C, and another set each of the low concentration and high concentration were kept at 37°C. The low and high temperatures were chosen to represent drinks kept in refrigerators and tropical climates, respectively. A pilot study was initially performed to approximate the survival time of both organisms in all drinks at both temperatures at 1 hour and 1 day. For the organism that did not survive up to 1 hour, the study was repeated and survival of organisms was reevaluated at 1, 3, 5, 15, 30, and 60 minutes. For the organism that survived up to 1 hour but not up to 1 day, survival of organisms was reevaluated at 1, 2, 4, 6, 8, and 24 hours. For the organism that survived up to 1 day, survival of organisms was reevaluated at 1, 3, 5, 7, 10, 12, 14, 21, 28, and 35 days. The study was performed in duplicate.

The survival and colony count of *B. pseudomallei* from beverages at the high concentration was assessed by collecting an aliquot of 100 μL and making four 10-fold dilutions with sterile distilled water (from 1:100, 1:1,000, and 1:10,000 to 1:100,000). Then an aliquot (100 μL) of each 10-fold dilution was spread on a whole Columbia agar plate using a rotary plater. For *B. pseudomallei* from beverages at the low concentration, an aliquot of 100 μL was dropped and spread directly on a whole Columbia agar plate. Another 100 μL from each beverage was directly inoculated into 3 mL of Tryptic soya broth and incubated in air at 37°C for 24 hours; consequently, an aliquot of 10 μL of surface broth was streaked onto a half of the Columbia agar plate. All Columbia agar plates were incubated in air at 37°C and inspected daily. A colony count was performed on day 4. The survival and colony count of *Leptospira* was assessed by spreading an aliquot of 100 µL onto a whole LVW agar plate. All LVW agar plates were incubated at 30°C in CO_2_ for 2 days and then in air at 30°C for 4 weeks in total. Colony counts were performed weekly for 4 weeks with the naked eye.^11^ Another 100 µL was inoculated into 1 mL of EMJH broth and incubated at 30°C. Survival of *Leptospira* in EMJH was detected by using a dark-field microscope weekly for 4 weeks.

Each beverage was evaluated for pH at 4°C, 25°C, and 37°C using pH meters (Mettler Toledo, Greifensee, Schweizerland). Univariable and multivariable Cox proportional hazard models were used to evaluate the conditions associated with time to culture negative of the organisms.

We found that time to culture negativity was different among beverages (*P* < 0.001). The shortest duration of survival was observed in Coca-Cola for both organisms in all conditions (Figures 1 and 2). We found that *B. pseudomallei* survived longer than *Leptospira* in all conditions (*P* < 0.001; Table 1). Both organisms survived longer in the high concentration (1 × 10^7^ CFU/mL) than the low concentration (1 × 10^3^ CFU/mL) (*P* = 0.001; Table 1). We found that both organisms survived longer in the four beverages at 4°C than at 37°C except in distilled water, in which both organisms survived for a shorter duration at 4°C than at 37°C. There was no difference in survival times between strains of *B. pseudomallei* (*P* > 0.99) and between strains of *Leptospira* (*P* > 0.99) evaluated.

**Figure 1.**
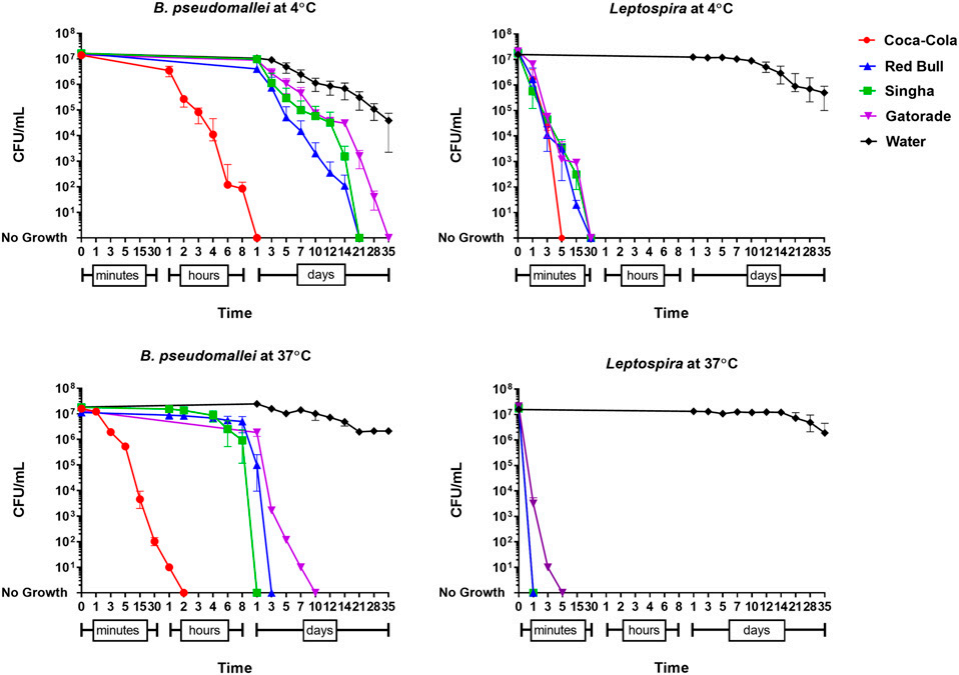
Survival of *Burkholderia pseudomallei* and pathogenic *Leptospira* in Coca-Cola, Red Bull, Singha beer, Gatorade, and distilled water at the high final concentration (1 × 10^7^ CFU/mL). This figure appears in color at www.ajtmh.org.

**Figure 2.**
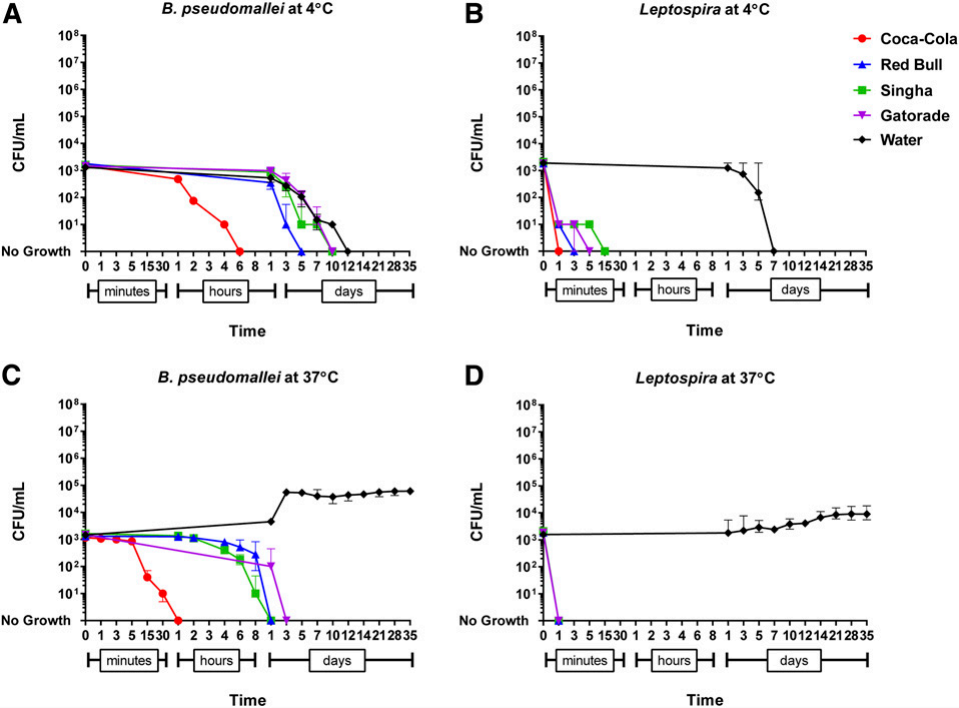
Survival of *Burkholderia pseudomallei* and pathogenic *Leptospira* in Coca-Cola, Red Bull, Singha beer, Gatorade, and distilled water at the low final concentration (1 × 10^3^ CFU/mL). This figure appears in color at www.ajtmh.org.

**Table 1:** Factors associated with time to culture negative of *Burkholderia pseudomallei* and pathogenic *Leptospira* in beverages and distilled water

Condition*	Crude hazard ratio (95% CI)	*P*-value	Adjusted hazard ratio (95% CI)	*P*-value
Organism				
Pathogenic *Leptospira*	1.0	< 0.001	1.0	< 0.001
*B. pseudomallei*	0.42 (0.28–0.63)		0.01 (< 0.01–0.05)	
Final concentration				
Low (1 × 10^3^ CFU/mL)	1.0	0.05	1.0	0.001
High (1 × 10^7^ CFU/mL)	0.67 (0.45–1.00)		0.46 (0.29–0.72)	
Beverages				
Distilled water	1.0	< 0.001	1.0	< 0.001
Coca-Cola	24.1 (9.3–62.5)		2030.1 (346.5–11,892.1)	
Red Bull	11.2 (4.5–27.7)		600.7 (115.8–3,115.2)	
Singha beer	10.2 (4.1–25.3)		512.1 (98.6–2,660.4)	
Gatorade	7.7 (3.1–19.3)		354.9 (69.5–1,811.3)	
Temperature				
37°C	1.0	0.02	1.0	0.01
4°C	0.58 (0.45–1.00)†		0.54 (0.33–0.87)	

CFU = colony-forming units; HR = hazard ratio.

* Hazard ratio represents the ratio of time to culture negative compared with the baseline condition (for which HR = 1.0) over time. An HR value of less than 1.0 indicates the time to culture negative was longer than the baseline condition, and vice versa.

† Excluding distilled water from the univariable analysis because both organisms survived in four beverages longer at 4°C than at 37°C except distilled water, in which both organisms survived for shorter duration at 4°C than at 37°C (Figures 1 and 2).

At 4°C and at the higher inoculum concentration, pathogenic *Leptospira* survived briefly in Coca-Cola, Red Bull, Singha, and Gatorade (up to 3, 15, 15, and 15 minutes, respectively; Figure 1B). By contrast, *B. pseudomallei* survived in these beverages in the same condition for up to 8 hours, 14 days, 14 days, and 28 days, respectively (Figure 1A). At 37°C, *Leptospira* spp. died in all four beverages within 5 minutes (Figures 1D and 2D).

The pH of Coca-Cola, Red Bull, Singha beer, Gatorade, and distilled water evaluated was 2.71, 3.72, 4.46, 3.37, and 6.98 (at 4°C); 2.50, 3.56, 4.30, 3.13, and 6.85 (at 25°C); and 2.44, 3.51, 4.26, 3.08, and 6.49 (at 37°C), respectively.

In this study, we demonstrated that pathogenic *Leptospira* organisms can survive in some beverages for up to 15 minutes, whereas *B. pseudomallei* can survive in some beverages for up to 4 weeks, particularly when the beverages are kept in a refrigerator at 4°C. This shows that people can get infected from contaminated cold drinks. A previous case report from Belgium found that drinking a can of soft drink with dried rat’s urine contaminating the top of the can could be the cause of leptospirosis and suggested washing cans before consumption.^14^ Presumably, the amount of bacteria contaminating the outside of the containers in those cases could not be killed by cold drinks within seconds of drinking. To reduce the risk of getting infections, we strongly suggest that people should always inspect containers of the drink or water; make sure that they have no holes, tears, or openings; and wash them carefully before drinking.

People should not be frightened by potential fake news about poor storage of drinks or deliberate contamination of the top of the can with bacteria or rat’s urine. *B. pseudomallei* is a Tier 1 select agent and controlled by local and international regulations.^2^ *Leptospira* spp. is difficult to grow and maintain and available only in a limited number of laboratories worldwide.^5^ The prevalence of captured rats being infected with leptospirosis is low even in leptospirosis-endemic areas.^7–9^ Therefore, the chance of deliberate release is low. For any potential fake news in the future, people should look for rumour-countering warnings, and expert organizations should correct any misinformation released.^15,16^ Nonetheless, the risk of accidental occurrence is not zero.

It is possible that the main factors in beverages associated with shorter duration of survival is the degree of acidity. Coca-Cola has the lowest pH and is associated with the shortest duration of survival in both organisms. This is supported by the previous studies showing that *B. pseudomallei* can survive in normal saline at pH 2.0 for 1 day and at 3.0 for 7 days,^17^ and *Leptospira* spp. is commonly observed in soil and water with pH around 5.5 and 7.6.^18^ The relatively shorter duration of *B. pseudomallei* survival at 37°C in Coca-Cola (less than 2 hours; pH 2.44) and in Red Bull and Singha beer (less than 3 days; pH from 3.51 to 4.26) observed in this study could be due to other ingredients such as preservatives, chemical additive, phosphoric acid, caffeine, and sodium citrate in the drinks.^19–21^

The longer survival time of *B. pseudomallei* than that of pathogenic *Leptospira* spp. is likely to be because *B. pseudomallei* is an environmental organism, whereas the main reservoirs of pathogenic *Leptospira* are rodents. Therefore, pathogenic *Leptospira* are less equipped to survive in a wide range of conditions of soil and water than *B. pseudomallei.*^2,3^

It is interesting that both organisms survive longer at 4°C than at 37°C in all four beverages but shorter at 4°C than at 37°C in distilled water. At lower temperature, cell composition, chemical reactions, membrane lipid fluidity, proteins, growth phase, growth rates, and other factors may change.^22^ Those factors may increase survival time in the extreme conditions in the beverages but might not be able to support the growth of the organisms in distilled water at 4°C compared with tropical climates at 37°C.

Our study has few limitations. We used culture to identify the survival of both organisms. We could not isolate the organisms at very low concentrations (e.g., < 10 CFU/mL) or as viable but non-culturable cells (VBNCs).
